# Serotonin Syndrome Presenting with Concomitant Tramadol and Diphenhydramine Use: A Case Report of an Unlikely Side-Effect

**DOI:** 10.7759/cureus.2421

**Published:** 2018-04-04

**Authors:** Salman Khan, Shakir Saud, Imran Khan, Muhammad Asif, Osama Ismail, Arqam Salam, Tsu Jung Yang, Kim J Norville

**Affiliations:** 1 Internal Medicine, Guthrie Clinic/Robert Packer Hospital, Sayre, USA; 2 Department of Cell Biology and Molecular Medicine, Rutgers New Jersey Medical School; 3 Department of Cardiology, Hofstra North Shore-LIJ School of Medicine, North Shore University Hospital; 4 Pulmonary/critical Care Medicine, Guthrie Clinic/Robert Packer Hospital, Sayre, USA

**Keywords:** serotonin

## Abstract

Serotonin syndrome is a condition that occurs following the administration of serotonergic drugs. Interestingly, on rare occasions, it can occur with various drug combinations that can secondarily affect the serum levels of 5‐hydroxytryptamin. Tramadol is an analgesic that has mu opioid receptor agonist activity and has also been shown to inhibit the reuptake of serotonin and noradrenaline. Diphenhydramine is a first-generation histamine antagonist prescribed to treat or prevent allergic reactions and can also be used as a sleeping aid. Here, we demonstrate a case of serotonin syndrome following the administration of diphenhydramine for seasonal allergies in a patient on tramadol for neck pain.

## Introduction

Serotonin syndrome is a rare and potentially life-threatening condition resulting from increased central nervous system (CNS) serotonergic activity, usually due to the concomitant use of certain medications. Selective serotonin reuptake inhibitors (SSRIs), serotonin and norepinephrine reuptake inhibitors (SNRs), tricyclic antidepressants, and monoamine oxidase inhibitors (MAOIs) have all been demonstrated to cause serotonin syndrome; however, in recent years, other drug classes have been increasingly implicated in serotonin toxicity.

## Case presentation

The patient is a 66-year-old man with hypertension, non-insulin-dependent diabetes mellitus type 2, seasonal allergies, and a recent traumatic injury to the head that occurred 20 days prior to admission, resulting in magnetic resonance imaging (MRI)-confirmed C5 superior facet fracture deemed non-surgical. The patient was subsequently prescribed tramadol, 50 mg tablets, as needed for symptomatic pain relief. On day one of his prescription, he took 100 mg tramadol. On day 2 of his prescription, he took 50 mg of tramadol. In addition, on day 2, he experienced a flare of seasonal allergies and took two tablets of 25 mg of diphenhydramine. Approximately 30 minutes after administration of the diphenhydramine, he experienced a sudden onset of spontaneous tongue fasciculations and tongue protrusion in a random, abnormal, semi-rhythmic manner that he recorded using his cell phone. He also had perioral twitching of the muscles and developed dystonic movements. He noticed his tremors were diffuse and symmetric with synchronous systemic contractions of the upper and lower extremities. He also noticed the movements to be episodic, becoming more frequent, as he never had this episode before. His family noted that he became increasingly confused, which prompted them to bring the patient to the emergency room.

**Figure 1:**
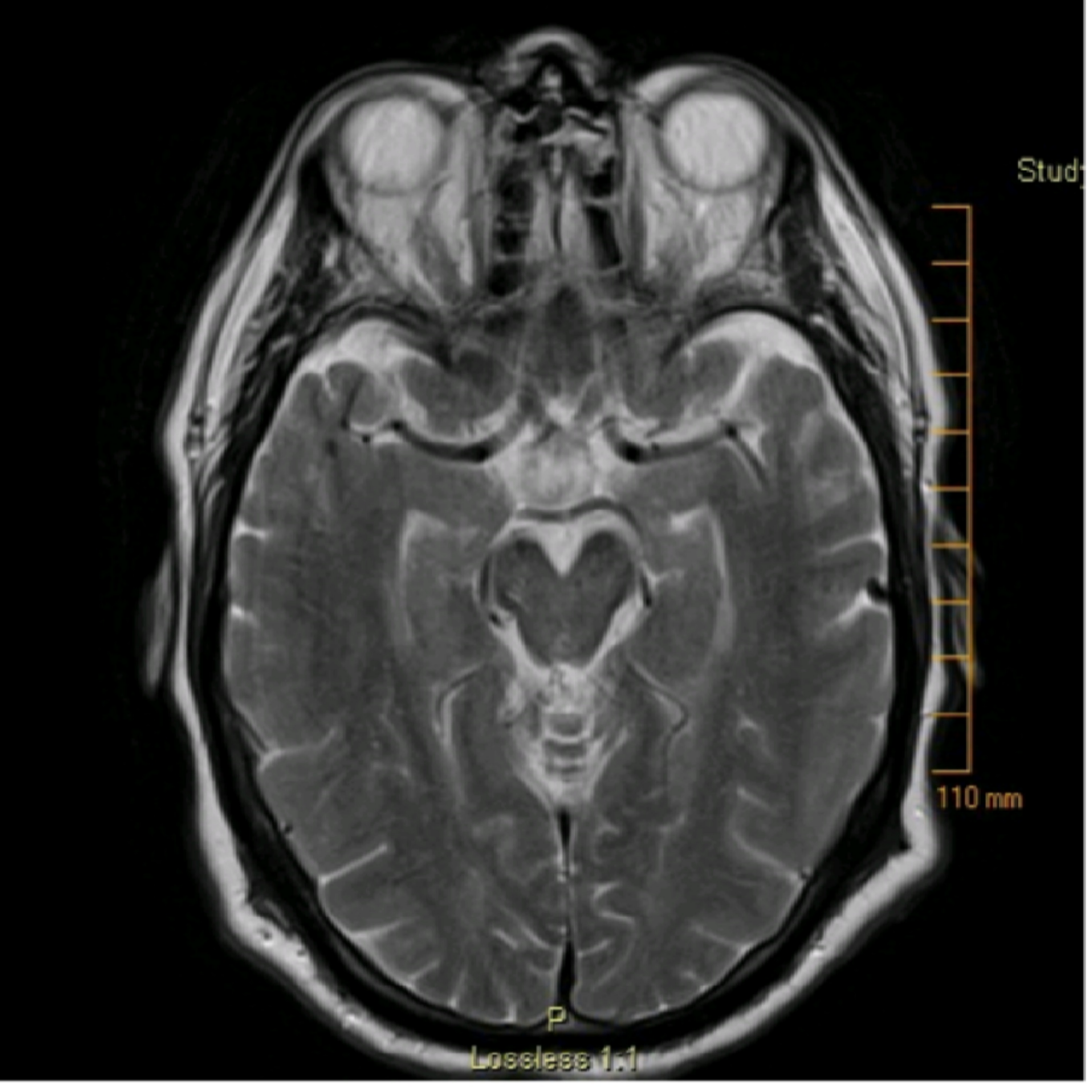
T2 Axial MR Brain MR: magnetic resonance

On presentation, the patient was increasingly confused, disoriented, and agitated. On exam, the patient was tachycardic, hypertensive, and tachypneic with a heart rate of 102, blood pressure of 200/100 mmHg, and respiratory rate of 22 breaths per minute, respectively, and was afebrile, with a temperature of 97.3 degrees Fahrenheit. Significant myoclonus and a non-focal neurological examination with hyperreflexia with clonus, particularly in the lower extremities, were present. Laboratory results were unrevealing. MRI of the brain without contrast revealed no evidence of acute ischemic changes and the magnetic resonance angiography (MRA) was unremarkable (Figure 1). Serotonin syndrome was a diagnosis of exclusion without evidence of an underlying metabolic, infectious, or cerebrovascular cause. Diphenhydramine and tramadol were discontinued and the patient was treated with intravenous (IV) fluids and IV alprazolam. No cyproheptadine was administered. Within 24 hours of admission, the patient clinically improved. He was alert and oriented and the myoclonus and hyperreflexia improved. The patient was subsequently transitioned to oral alprazolam and discharged the following day with follow-up with neurology. This case presents a rare incidence of serotonin syndrome occurring secondary to a drug interaction of otherwise benign medications.

## Discussion

Serotonin syndrome is a rare and potentially life-threatening condition resulting from increased CNS serotonergic activity usually due to the concomitant use of certain medications. Selective serotonin reuptake inhibitors (SSRIs), serotonin and norepinephrine reuptake inhibitors (SNRs), tricyclic antidepressants, and monoamine oxidase inhibitors (MAOIs) have all been demonstrated to cause serotonin syndrome; however, in recent years other drug classes have been increasingly implicated in serotonin toxicity.

Tramadol is a synthetic analog of codeine that exerts analgesic effects by acting primarily on the opioid mu receptor [1]. Tramadol, with its lower risk of addiction and overall safety profile, is generally considered safe medication and efficacious in providing pain relief as compared to other opioids. Interestingly, it is thought that tramadol’s analgesic effect is due to the inhibition of serotonin and norepinephrine reuptake in the CNS [2] and the risk of causing serotonin syndrome is currently well-recognized. Diphenhydramine, a first-generation antihistamine that acts as an inverse agonist on the H1 receptor [3] may also inhibit the reuptake of serotonin. It is known that SSRIs like Fluoxetine are analogs of diphenhydramine [4]. Although weaker, diphenhydramine does retain some activity at the serotonin receptor. In pre-clinical models, it was shown that by acting as a completive antagonist at the muscarinic acetylcholine receptor, diphenhydramine inhibits post-synaptic reuptake of serotonin, which is estimated a sixty-four percent in the presence of narcotics [5]. Taken together, in conjunction with another serotonergic drug, the otherwise weak activity of diphenhydramine at the receptor could result in serotonin syndrome, as in the present case.

Serotonin toxicity results from the over-activation of peripheral and central postsynaptic serotonin receptors, particularly 5-HT 1A, 2A, and 3 [6]. Once stimulated, there is a combination of mental status changes, neuromuscular hyperactivity, and autonomic instability that usually manifest within 24 hours. In mild cases, patients present with mild hypertension and tachycardia with some combination of mydriasis, diaphoresis, shivering, tremor, myoclonus, and hyperreflexia. In most cases, patients with a mild syndrome are afebrile, as in the present case. Moderate cases, in addition to the above symptoms, would present with hyperthermia, clonus, agitation, hypervigilance, and/or pressured speech are present. In severe cases, hyperthermia is greater than 41.1°C with delirium and muscle rigidity. Severe cases may result in coma and death by many complications, such as seizures, rhabdomyolysis, myoglobinuria, metabolic acidosis, renal failure, acute respiratory distress syndrome, respiratory failure, and diffuse intravascular clotting [7].

Serotonin syndrome can be diagnosed using the Hunter Serotonin toxicity criteria [8]. The diagnosis is made with the presence of one of the following features: spontaneous clonus; inducible clonus with agitation or diaphoresis; ocular clonus with agitation or diaphoresis; tremor and hyperreflexia; or hypertonia, temperature above 100.4° F (38° C), and ocular or inducible clonus. In the present case, the patient presented with mild-moderate symptoms with hypertension and tachycardia with ocular and inducible clonus, agitation, diaphoresis, tremor, and hyperreflexia.

Management consists of discontinuing the offending agent and providing supportive care, which includes intravenous fluids and the use of benzodiazepines for the alleviation of symptoms. Signs and symptoms typically resolve within 24 hours after the discontinuation of the causative medication, as in the present case and shown in previous case reports [9]. Serotonin antagonists, such as cyproheptadine, can be used in moderate to severe cases or if symptoms persist, resulting in symptom relief within one to three days [10].

## Conclusions

The present case is consistent with a diagnosis of mild to moderate serotonin syndrome. While considered rare, the growing number of serotonergic drugs that are available in clinical practice is expected to increase the incidence of serotonin syndrome. Awareness of the present case, as well as other similar cases, is essential for the prevention of serotonin syndrome. Future studies should be aimed at identifying potential serotonergic interactions and minimizing the concomitant use of multi-drug regimens that could put patients at risk for developing serotonin syndrome.
